# Innovative Management of a Giant Fibroid in Pregnancy: A Case Report

**DOI:** 10.7759/cureus.70894

**Published:** 2024-10-05

**Authors:** Safina Tanveer, Tooba Shah, Faiza Gul, Fahim Ullah, Aqsa Tariq

**Affiliations:** 1 Surgery, Khyber Teaching Hospital, Peshawar, PAK; 2 Obstetrics and Gynecology, Lady Reading Hospital, Peshawar, PAK; 3 Pediatrics, Northwest General Hospital, Peshawar, PAK; 4 Cardiac Surgery, Armed Forces Institute of Cardiology/National Institute of Heart Diseases, Rawalpindi, PAK; 5 Internal Medicine, Nishtar Medical University, Multan, PAK

**Keywords:** cutting-edge obstetric care, innovative surgical techniques, interval myomectomy strategy, patient-centered surgical solutions, uterine fibroid management

## Abstract

Uterine fibroids are the most common benign tumors of the female reproductive system, frequently found in women. Although typically asymptomatic during pregnancy, complications can arise in some cases. Management of fibroids during pregnancy often involves a conservative approach, with myomectomy considered after delivery. Alternatively, cesarean or antepartum myomectomy can be tailored according to the patient's needs. In this case report, we present a G2P1 patient who, during her second trimester, experienced increased abdominal girth, pelvic discomfort, constipation, and urinary incontinence. An early multidisciplinary team (MDT) consultation, involving obstetrics and gynecology, general surgery, and urology, identified a massive posterior fibroid as the cause of these obstructive and compressive symptoms. The patient was admitted for symptom relief and ultimately underwent an elective cesarean section at 35 weeks of gestation. The outcome was a live male baby weighing 2.5 kg. Two months later, an interval myomectomy was performed, successfully removing a massive fibroid measuring 22x17x10 cm and weighing 7 kg. While cesarean myomectomy is considered a safe procedure with no greater risk than a cesarean section alone and is also a cost-effective approach, it was not feasible in this case due to the fibroid’s proximity to major pelvic blood vessels.

## Introduction

Uterine fibroids are the most common benign tumors of the female reproductive system, affecting 20%-40% of women. Their estimated occurrence during pregnancy ranges from 0.1% to 3.9% [1]. While fibroids are hormone-dependent growths, their expansion during pregnancy appears to follow a nonlinear pattern [2]. Fibroids can occur as single or multiple growths, varying in size. During pregnancy, uterine fibroids are typically asymptomatic, though complications arise in 10%-30% of cases [1]. Therefore, patients with uterine fibroids during pregnancy should receive thorough counseling regarding the potential risks and adverse outcomes [3].

The primary outcomes associated with pregnancy in the presence of at least one leiomyoma include intrauterine fetal death, breech presentation, placenta previa, placental abruption, preeclampsia, fetal growth restriction, preterm premature rupture of membranes, cesarean delivery, and preterm birth [4]. Multiple fibroids, however, do not appear to increase the risk of these complications. Larger fibroids, on the other hand, are significantly associated with a higher risk of breech presentation, postpartum hemorrhage (PPH), and placenta previa compared to smaller fibroids [5]. The primary approach to managing uterine fibroids during pregnancy is conservative, with counseling for a possible myomectomy after delivery. Antepartum myomectomy or cesarean myomectomy have also been successfully performed in selective cases [1].

This case demonstrates the morbidity associated with a 14 cm leiomyoma during pregnancy and highlights the essential role of multidisciplinary coordination between the Obstetrics and Gynecology and General Surgery teams in managing complex cases of large uterine fibroids during gestation and postpartum period. Such collaborative care is crucial in optimizing maternal and fetal outcomes.

## Case presentation

This case report details the multidisciplinary management of a 32-year-old gravida two, para one female, with a history of delivering a healthy male child, presented at 25 weeks of gestation with a large uterine leiomyoma. The patient, with no known comorbidities, complained of increased abdominal girth, disproportionate pelvic discomfort, constipation, and urinary incontinence. She reported that her symptoms had started during the second trimester and progressively worsened. Her past medical history was insignificant, except for a previous cesarean section due to a sub-serosal fibroid located close to the cervix. Moreover, between the two pregnancies, the fibroid remained silent, and the patient did not undergo any treatment as it was asymptomatic. It caused no issues during the first pregnancy as well. 

**Figure 1:**
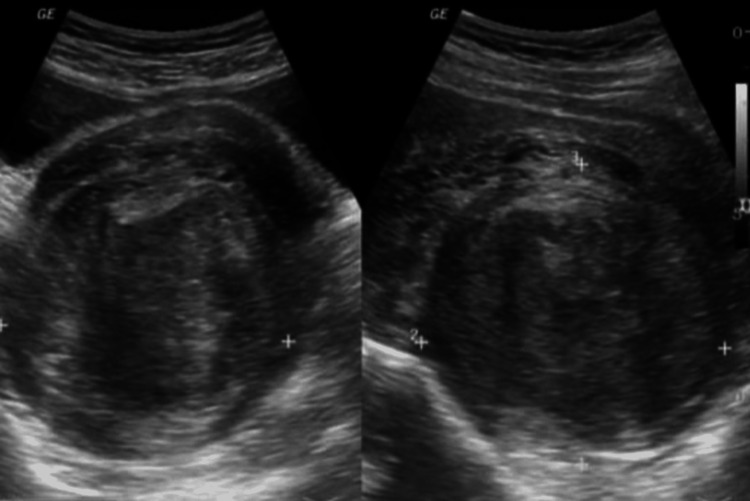
Subserosal fibroid located on the posterior uterine wall compressing the uterine cavity, complicating fetal diagnosis.

On physical examination, her abdomen was grossly distended, and nontender, with a large, firm abdominopelvic mass. Routine investigations and abdomino-pelvic ultrasound confirmed, a massive sub-serosal posterior fibroid measuring 14 cm × 18 cm, extending into the rectovesical pouch, compressing the rectum, sigmoid colon, left ureter, and bladder (Figure 1).

Due to the complex nature of her case, a multidisciplinary team (MDT) meeting involving OBGYN, general surgery, and urology specialists was held. The discussion weighed the risks of continuing the pregnancy with the large fibroid against the potential complications of preterm delivery, including miscarriage, hemorrhage, intestinal obstruction, and urinary tract infections. It was agreed in the MDT to implement more frequent antenatal monitoring, with the general surgery team consistently involved for close follow-up. The team also discussed two options with the patient: a planned elective cesarean section at 34 weeks or a watchful waiting approach with the possibility of emergency surgery. After thorough counseling, the patient opted for an elective cesarean section at 34 weeks.

The patient was seen at two-week intervals in the antenatal period, with reviews by the general surgery and urology teams. Intramuscular dexamethasone was administered to promote fetal lung maturity. At 35 weeks of gestation, an elective cesarean section was performed under spinal anesthesia with the surgery team on standby. A Pfannenstiel incision was made and a routine entry into the peritoneal cavity was made. The presence of a large fibroid uterus was apparent, with the largest fibroid causing significant distortion of the normal pelvic anatomy and uterine angles. To minimize the risk of injuring major blood vessels and fibroid tissue, a lateral-transverse incision was deemed the most appropriate surgical approach. A healthy male infant weighing 2.5 kg was delivered with Apgar scores of 5 and 8 at one and five minutes, respectively. Due to the large posterior fibroid, uterine contractility was significantly altered, leading to an estimated blood loss of approximately 800 mL. Therefore, the myomectomy was postponed. Hemostasis was secured, and uterotonics, including oxytocin, tranexamic acid, and carboprostol, were administered. The uterine incision was repaired without exteriorizing the uterus, and the patient recovered uneventfully. She was discharged after three days. The infant was hospitalized for five days and was in overall good health. He was monitored until he established stable feeding.

**Figure 2:**
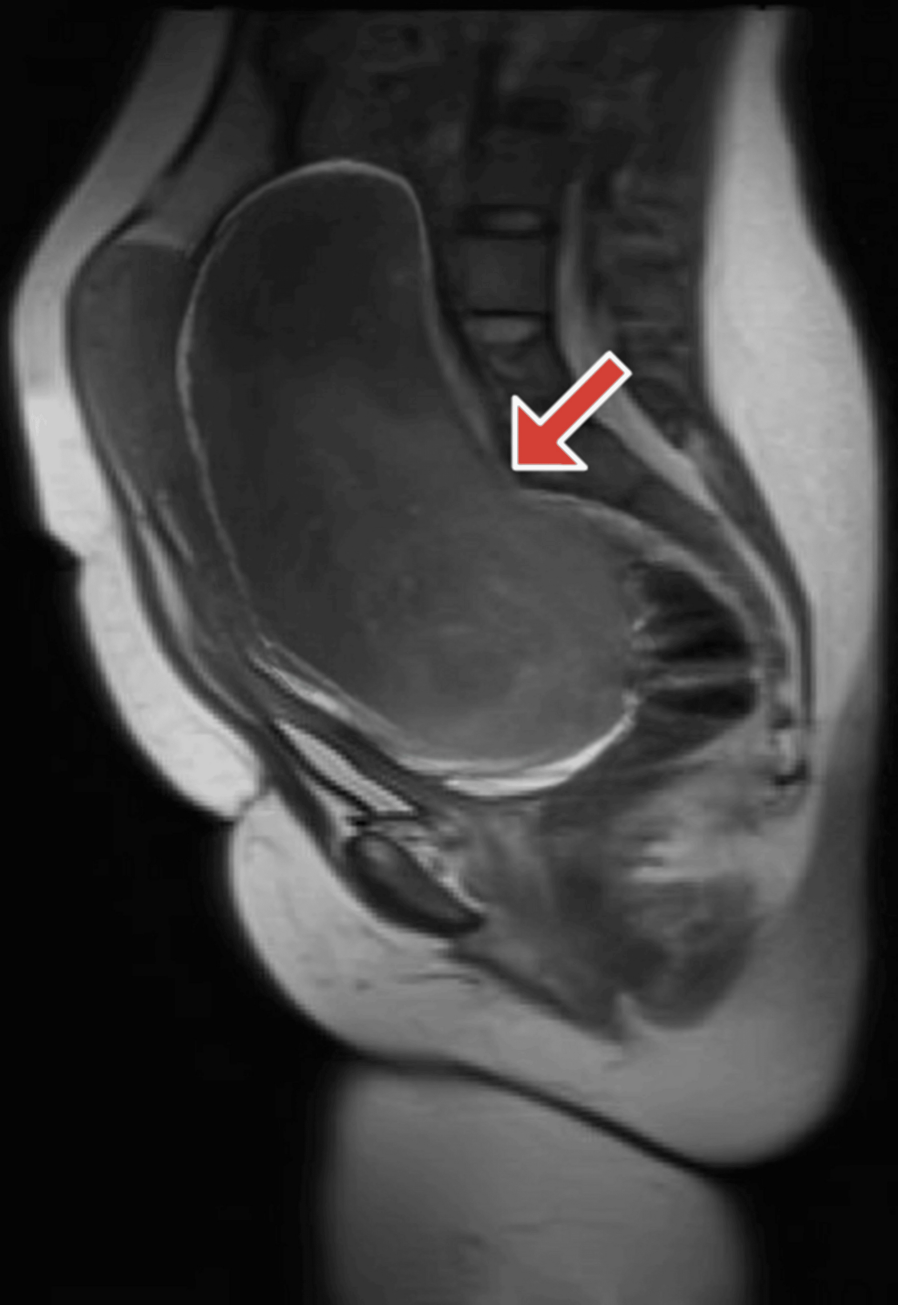
Post-puerperium MRI pelvis with contrast, 10 weeks later, reveals a well-defined oval-shaped pelvic mass compressing the uterus anteriorly and displacing both the rectum and bladder.

Six weeks postpartum, the patient returned with persistent pelvic discomfort and pain. An MRI of the pelvis revealed a huge about 20x17x10 cm, well-defined oval-shaped pelvic mass compressing the uterus anteriorly and displacing the rectum and bladder. The mass extended from the lower posterior uterine wall, with indistinct margins, and no cystic or necrotic areas were noted. Following delivery, a reduction in fibroid size was anticipated due to the drop in estrogen and progesterone levels. However, instead of regressing, the fibroid increased in size from 14x18 cm during pregnancy to 22x17 cm x10cm, as confirmed by MRI 10 weeks postpartum (Figure 2).

During the surgery, a midline incision was made, and the peritoneal cavity was entered. The massive fibroid, which distorted the pelvic anatomy, was located posteriorly. An intracapsular myomectomy, without breaching the endometrial cavity, was successfully performed with the application of a Foley catheter tourniquet at the level of the internal cervical os. The fibroid, weighing 7 kg, was removed intact and sent for histopathology.

**Figure 3:**
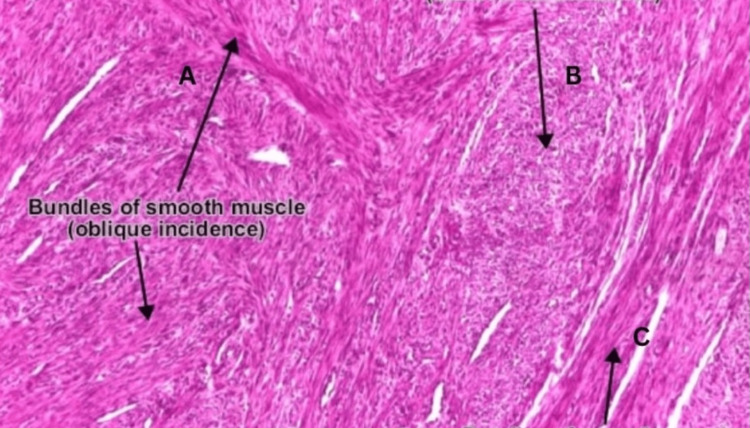
Microscopic examination of the fibroid specimen using hematoxylin and eosin stain at 100x magnification revealed a tumor composed of bland-looking spindle cells arranged in oblique (A), transverse (B), and longitudinal (C) patterns. No evidence of necrosis or malignancy observed.

The histopathological examination, performed using hematoxylin and eosin staining and examined at 100x magnification, revealed a tumor composed of bland-looking spindle cells arranged in fascicles. There was no evidence of increased mitosis, necrosis, nuclear atypia, or malignancy. The histopathological examination confirmed a leiomyoma (Figure 3).

The surgery involved significant blood loss, amounting to 1500 mL, which required a whole blood transfusion. Despite this, intensive care was not needed as the patient remained hemodynamically stable upon recovery from anesthesia. Postoperatively, the patient recovered well with the administration of analgesics and antibiotics and was discharged five days later. At the six-week postoperative follow-up, the patient was stable and free from her initial symptoms, marking a successful outcome to a challenging case.

## Discussion

This case involves a high-risk pregnancy complicated by the presence of a large uterine fibroid, which caused obstructive symptoms during the second trimester. Early involvement of an MDT, including specialists from OBGYN, general surgery, and urology, was crucial to ensure the best possible outcome for both the patient and the fetus while minimizing the risks associated with pregnancy and delivery. 

Cesarean myomectomy was deemed unsafe in this case, as the imaging workup during pregnancy revealed that the fibroid was located near major pelvic blood vessels, increasing the risk of significant intra and postoperative hemorrhage. The patient declined the option of a cesarean hysterectomy because she hoped to have more children in the future, so it was not considered. Comprehensive counseling played a key role in ensuring the patient’s commitment to undergoing a myomectomy after the baby was delivered. As a result, an interval myomectomy was performed after delivery. This case is reported for its unique and complex clinical management.

Uterine fibroids are the most frequent benign tumors found in women, affecting around 20% to 30% of those in their reproductive years. As such, they are often encountered during pregnancy. However, the exact frequency of fibroids in pregnancy remains unclear, with reported incidence rates ranging from as little as 0.1% of all pregnancies to as high as 12.5% [6]. Fibroids during pregnancy are gaining increased importance in modern obstetrics due to several factors, including a demographic trend towards delayed childbearing, the growing prevalence of obesity, and the increasing number of pregnancies following fibroid treatment [7].

Various risk factors, both modifiable and non-modifiable, contribute to the development of fibroids. These include age, race, hormonal influences (both endogenous and exogenous), obesity, uterine infections, and lifestyle factors such as diet, caffeine and alcohol intake, physical activity, stress levels, and smoking [8]. Most fibroids are small (< 5 cm) and remain stable in size. While approximately 30% of fibroids enlarge due to hormonal changes during pregnancy, however, they usually regress postpartum [9]. In this case, instead of the expected regression, the fibroid increased in size from 14 x 18 cm to 22 x 17 cm. 

The primary complication associated with fibroids during pregnancy is the painful myoma syndrome, which is marked by pain during the second and early third trimesters, sometimes accompanied by bleeding. Additional complications include preterm premature rupture of membranes, abnormal fetal presentation, higher rates of cesarean delivery, and postpartum endomyometritis [10]. Fibroids located posteriorly and larger than 30 mm in diameter tend to cause more severe pelvic pain compared to those located anteriorly [11].

The development of any of the above complications during pregnancy may necessitate abandoning the typical conservative approach to managing uterine fibroids. The decision to perform a myomectomy during pregnancy requires careful consideration due to the significantly increased uterine vascularization. Performing a myomectomy in this context carries a high risk of excessive blood loss, which could result in the need for an emergency hysterectomy or even maternal mortality [1].

An alternative approach is cesarean myomectomy. A study comparing two groups - one with uterine fibroids undergoing cesarean myomectomy and another without fibroids undergoing standard cesarean section - found no significant differences in the need for blood transfusion, postoperative febrile morbidity, or hospital stay duration between the groups. These findings suggest that cesarean myomectomy is a safe procedure, posing no greater risk to patients than a cesarean section alone. This approach also benefits the healthcare sector by eliminating the need for an interval myomectomy, thereby enhancing cost-effectiveness [12]. The management of large subserosal fibroids is influenced by symptoms, patient preferences, and fertility considerations. Surgical options include hysterectomy for those not wishing to preserve fertility and myomectomy for those who do, though the latter carries a 15% to 30% recurrence risk within five years. Uterine artery embolization (UAE) is a minimally invasive alternative that shrinks fibroids but is approached with caution in patients desiring future pregnancies due to potential complications. Non-surgical treatments, such as GnRH agonists, can temporarily alleviate symptoms but are not meant for long-term use, while MRI-guided focused ultrasound offers a non-invasive method to target fibroids. Ultimately, management should be individualized based on the patient's specific circumstances and needs, with asymptomatic cases often managed through watchful waiting​ [13].

In our case, the patient responded well to conservative management, so antepartum myomectomy was not performed. Cesarean myomectomy was also ruled out due to the fibroid's proximity to major pelvic vessels, which posed a higher risk of significant intraoperative hemorrhage. Interval myomectomy was considered a safe option for this patient, as it was anticipated that the fibroid size would decrease, making the procedure easier with reduced risk of bleeding. As a result, an interval myomectomy was planned instead.

## Conclusions

This case highlights that conservative management followed by interval myomectomy is a safe and effective approach for managing high-risk pregnancies complicated by large uterine fibroids. While cesarean myomectomy offers cost-effectiveness by eliminating the need for a later myomectomy, individualized strategies focused on reducing maternal morbidity and mortality are of greater importance. Early involvement of an MDT also contributed to the successful outcome.
